# MYIASE GASTRO-INTESTINALE À *DROSOPHILA MELANOGASTER* : À PROPOS D'UN CAS MAROCAIN

**DOI:** 10.48327/mtsi.v3i3.2023.360

**Published:** 2023-07-10

**Authors:** Zaineb HAMMOUCH, Asmae EL ASSIL, Mohamed LYAGOUBI, Sara AOUFI

**Affiliations:** 1 Laboratoire central de parasitologie et mycologie, Hôpital Ibn Sina, 10170 Rabat, Maroc; 2 Faculté de médecine et de pharmacie, Université Mohammed V, 10170 Rabat, Maroc

**Keywords:** Myiase gastro-intestinale, *Drosophila melanogaster*, Brachycères, Parasitologie, Maroc, Maghreb, Afrique du Nord, Gastrointestinal myiasis, *Drosophila melanogaster*, Brachycetes, Parasitology, Morocco, Maghreb, Northern Africa

## Abstract

**Introduction:**

Les myiases sont des infestations des humains ou des animaux par des formes larvaires des brachycères. Les localisations fréquemment observées sont otorhino-laryngologiques et cutanées. La localisation gastro-intestinale reste exceptionnelle. Nous rapportons un cas marocain de myiase gastro-intestinale à *Drosophila melanogaster*. Observation. Il s'agit d'un patient âgé de 56 ans vivant dans une région rurale du nord-ouest du Maroc. Il était suivi à l'Institut national d'oncologie de Rabat pour adénocarcinome pulmonaire et rénal, et mis sous chimiothérapie, néo-adjuvante avec la radiochimiothérapie concomitante. Le patient a présenté des vomissements à deux reprises contenant une quarantaine de petits vers blanchâtres de 4 mm de longueur à section circulaire et glabres. Ce prélèvement a été adressé au laboratoire de parasitologie et mycologie pour identification. L’étude microscopique des larves et des mouches adultes, obtenues après élevage au laboratoire de parasitologie, a permis de diagnostiquer une myiase à *Drosophila melanogaster*.

**Discussion/Conclusion:**

Cette observation s'individualise par sa localisation anatomique qui reste exceptionnelle d'une part et par le genre du parasite incriminé d'autre part.

## Abstract (en)

**Introduction:**

Myiasis is an infestation of humans or animals by larval forms of brachycetes. Commonly observed locations are otolaryngological and cutaneous. Gastrointestinal localization remains exceptional. In this work, we report the observation of a Moroccan case of gastrointestinal myiasis caused by *Drosophila melanogaster*. Observation. The patient was a 56-year-old man living in a rural region of northwest Morocco. He was being followed at the National Oncology Institute in Rabat for pulmonary and renal adenocarcinoma and was put on neo-adjuvant chemotherapy with concomitant radiochemotherapy. The patient presented vomiting twice containing about forty small white, circular and hairless worms measuring 4 mm in length. This sample was sent to the parasitology and mycology laboratory for identification. Microscopic study of the larvae and adult flies obtained after rearing in the laboratory of parasitology allowed the diagnosis of myiasis caused by *Drosophila melanogaster*.

**Discussion/Conclusion:**

This observation is noteworthy for both its exceptional anatomical localization and the implicated parasite species.

## Introduction

Les myiases, dont le nom est dérivé du grec *myia* (mouche) et *iasis* (maladie), sont des infestations des humains ou des animaux par des formes larvaires des diptères brachycères [9]. Les localisations fréquemment observées sont oto-rhino-laryngologiques et cutanées. La localisation gastro-intestinale reste exceptionnelle [1].

Nous rapportons dans ce travail l'observation d'un cas marocain de myiase gastrointestinale à *Drosophila melanogaster.* Nous envisageons également à la lumière de la littérature, les aspects épidémiologiques, cliniques et diagnostiques de cette parasitose.

## OBSERVATION

**Figure 1:**
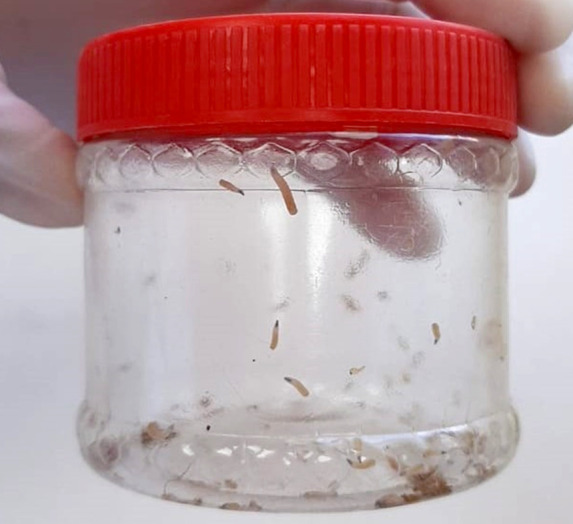
Prélèvement adressé au laboratoire de parasitologie et de mycologie de Rabat (crédit photo : Z. HAMMOUCH) *Sample addressed to the laboratory of parasitology and mycology of Rabat (photo credit:* Z. HAMMOUCH)

Il s'agit d'un patient âgé de 56 ans vivant dans une région rurale du nord-ouest du Maroc. Il était suivi à l'Institut national d'oncologie de Rabat pour adénocarcinome pulmonaire et rénal à cellules claires, et mis sous chimiothérapie, néo-adjuvante avec la radiochimiothérapie concomitante. Le patient a présenté des vomissements à deux reprises contenant une quarantaine de petits vers blanchâtres mobiles de 4 mm de longueur à section circulaire et glabres, avec absence d'autres signes digestifs. L’état général du patient était conservé. L'hémogramme a montré une hyperéosinophilie à 0,75 G/l. Ce prélèvement a été adressé au laboratoire de parasitologie et mycologie de Rabat pour identification (Fig. 1).

Dans le but d'identifier le parasite, nous nous sommes basés sur l'observation des vers émis par les vomissements, à la loupe et au microscope optique. Les larves étaient de couleur blanche à beige, de 3 à 4 mm de longueur et glabres. Elles se composaient d'une capsule céphalique [tête] rétractile, qui apparaissait à l'oeil nu comme un point noir, suivie de 13 segments.

**Figure 2:**
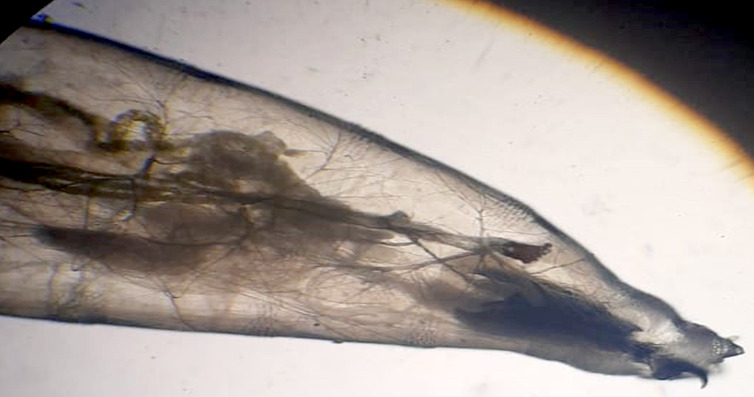
Partie antérieure [crochet + armature pharyngienne + stigmates respiratoires] (Grossissement x10) (crédit photo : Z. HAMMOUCH) *Anterior section [hook + pharyngeal armature + respiratory stigmas] (Magnification x10) (photo credit:* Z. HAMMOUCH)

En ce qui concerne l'extrémité antérieure de la larve, la capsule céphalique contenait un crochet buccal suivi par une pièce chitineuse, qui est l'armature pharyngienne, et par des stigmates respiratoires en forme d’éventail disposés latéralement (Fig. 2).

**Figure 3:**
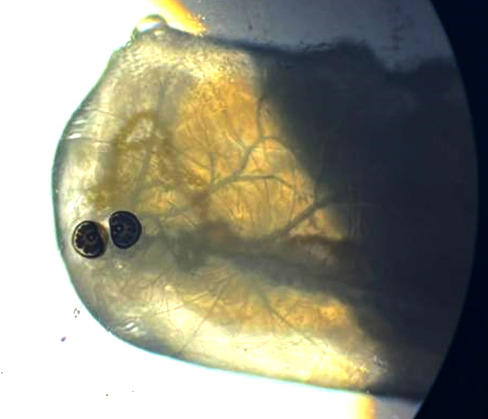
Stigmates respiratoires postérieurs (Grossissement x10) (crédit photo : Z. HAMMOUCH) *Figure 3: Posterior respiratory stigmas (Magnification x10) (photo credit:* Z. HAMMOUCH)

L'extrémité postérieure de la larve était constituée des stigmates respiratoires en forme de D, à l'intérieur desquels se trouvaient trois fentes stigmatiques dont l'aspect rappelait celui d'un cordon (Fig. 3).

Le résultat de l'examen parasitologique des vers émis avec les vomissements a conduit, avec la clé d'identification des diptères [6], à l'identification de *Musca domestica*. Dans le but de compléter et de confirmer l'identification du diptère incriminé dans cette myiase, nous avons procédé à l’élevage des larves émises par les vomissements. Celui-ci a été réalisé à température ambiante dans un flacon propre contenant les éléments suivants:

- au fond : un milieu nutritif composé d'eau, levure de bière, sucre et lait en poudre;
- au milieu : un support qui permet aux mouches de se poser en évitant l'engluement dans le milieu nutritif [compresses stériles];
- un bouchon qui doit laisser passer l'air [parafilm].

**Figure 4:**
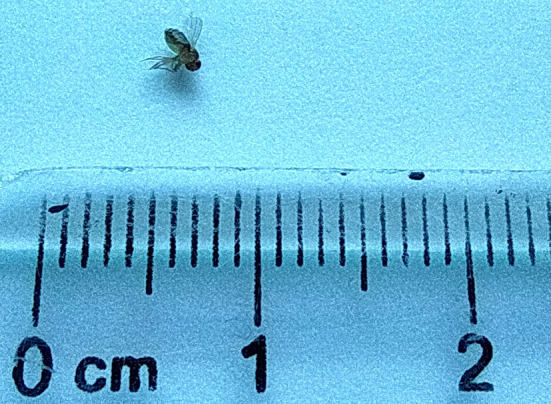
L'imago résultant observé à la loupe (crédit photo : Z. HAMMOUCH) *The resulting adult observed with a magnifying glass (photo credit:* Z. HAMMOUCH)

**Figure 5:**
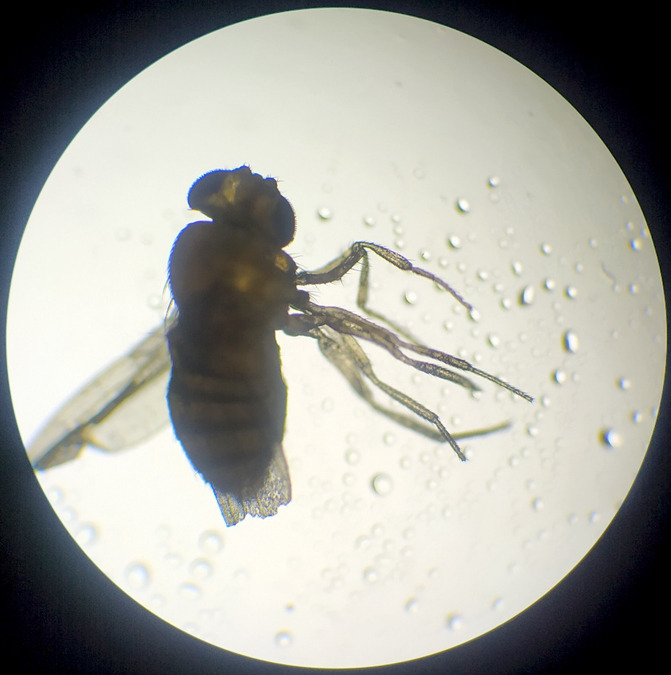
L'imago observé au microscope optique (Grossissement x10) (crédit photo : Z. HAMMOUCH) *The resulting adult observed with an optical microscope (Magnification x10) (photo credit:* Z. HAMMOUCH)

Après 3 jours d’élevage, nous avons obtenu la pupe que nous avons observée au microscope optique et à la loupe. Cette dernière se présentait sous la forme d'un tonnelet de couleur brun clair, et présentait des expansions de chaque côté du dernier segment. Après 3 jours du stade pupal, nous avons obtenu l'adulte (l'imago). Nous avons procédé ensuite à son identification en nous basant sur l'observation à la loupe (Fig. 4) et au microscope optique (Fig. 5) des différentes parties de son corps : yeux, antennes, abdomen, pattes et ailes.

Il s'agissait d'un diptère de 3 mm de long, de couleur jaune pâle avec des anneaux noirs transversaux sur l'abdomen, des yeux de couleur rouge brique, des antennes plumeuses, 3 paires de pattes et une paire fonctionnelle d'ailes hyalines sans taches.

Le résultat de l'examen parasitologique des adultes obtenus après élevage au laboratoire a permis, avec la clé d'identification des diptères [6], l'identification des adultes du genre *Drosophila melanogaster.*

## Discussion

Les diptères cyclorraphes responsables des myiases humaines se retrouvent dans toutes les régions du monde. Cependant, chaque espèce se cantonne à une région plus ou moins étendue du globe (*OEstrus*, *Cordylobia* en Afrique; *Dermatobia* en Amérique du Sud; *Wohlfahrtia, Hypoderma* en Europe) [7, 10]. Dans le contexte marocain, les myiases humaines sont déterminées essentiellement par deux familles. D'une part la famille des OEstridés, notamment *OEstrus ovis* qui est fréquente au sud du Maroc [1, 17]. Cette espèce est responsable des myiases principalement des cavités oto-rhino-laryngologiques. Les myiases cutanées sont provoquées par le genre *Hypoderma*, infestant le bétail (ouest du Maroc) [17]. D'autre part la famille des Sarcophagidés, et précisément *Wohlfahrtia magnifica,* est incriminée dans les myiases de l'oreille et de la crête nasale et dans des myiases à localisation gingivale [1, 12].

Les myiases gastro-intestinales sont exceptionnelles [1], dues essentiellement à *Drosophila, Calliphora, Lucilia* et *Sarcophaga* [3, 14]. Au Maroc, seuls deux cas ont été rapportés, dont l'identification parasitologique des vers a conclu à des larves de *Sarcophaga hemorrhoidalis* [1] et d’*Eristalis tenax* [14].

Toutefois, malgré la rareté des myiases gastro-intestinales, plusieurs arguments plaident en faveur de ce diagnostic dans le cas que nous rapportons. Ainsi, chez notre patient, aucun symptôme digestif n'a été initialement rapporté, hormis les deux épisodes de vomissements, survenus à distance des séances de chimiothérapie et de radiothérapie et marqués par l'expulsion d'un grand nombre de larves vivantes et mobiles. Qui plus est, l'observation d'une hyperéosinophilie marquée (0,75 G/l), ainsi que la constatation d'un soulagement des symptômes suite à l’élimination des larves constituent des arguments supplémentaires de l'implication de la myiase gastro-intestinale dans la survenue de ces manifestations cliniques.

Deux cas de myiases à *Drosophila melanogaster* ont été rapportés dans la littérature : un cas de myiase nasale chez un patient ayant visité la côte sud de la Turquie où la *Drosophila melanogaster* est endémique [2], et un cas de myiase de la région péri-orbitaire chez un nourrisson originaire d'Amérique du Nord [5].

Les larves et/ou les oeufs sont ingérés avec des aliments [fromage, légumes, fruits] et/ou de l'eau. Elles sont en général détruites dans le tube digestif par les sucs gastro-intestinaux. Parfois, elles résistent suffisamment longtemps et déterminent alors une symptomatologie digestive : des troubles gastriques à type de nausées, de violentes douleurs épigastriques et des vomissements évacuant les larves. Les signes intestinaux comportent des douleurs abdominales avec diarrhées, voire des hémorragies et même des perforations digestives [1, 16].

*Drosophila melanogaster* a été utilisée depuis de nombreuses années comme modèle pour des études génétiques et physiologiques. Malgré cela, la littérature ne contient pas suffisamment de descriptions détaillées des stades immatures de diptères, tels que *Drosophila melanogaster* [11]. Il existe de nombreuses similitudes anatomiques entre *Musca domestica* et *Drosophila melanogaster*, notamment l'absence d'ouvertures spiraculaires antérieures pendant le premier stade larvaire [11], des organes antennaires identiques [4], un système musculaire similaire du céphalosquelette [18], ainsi qu'un processus de métamorphose identique [15]. Ces similitudes soulignent les limites des clés d'identification basées sur la morphologie non détaillée des stades immatures des diptères myiasigènes, qui peuvent conduire à des erreurs de diagnostic, comme dans notre cas où une larve de *Musca* a été initialement identifiée comme telle, mais s'est avérée être une larve de *Drosophila* après son passage à l’état adulte.

Il n'existe pas de traitement spécifique pour la myiase gastro-intestinale. Les agents anti-inflammatoires et antipéristaltiques réduiront les symptômes, tandis que les anthelminthiques à large spectre et les antibiotiques ne seront pas utiles. Par conséquent, le meilleur traitement repose sur des mesures prophylactiques basées sur l'hygiène alimentaire, l’éducation sanitaire et la lutte contre l'agent vecteur de myiase par l'utilisation des insecticides. Le seul traitement efficace dans cette localisation gastro-intestinale se limite à des lavages gastriques utilisant des solutions isotoniques, associées à des laxatifs dans le but d’éliminer les larves. L'ivermectine a été essayée sans effet significatif [1,8,9,13].

## Conclusion

À notre connaissance, nous rapportons dans ce travail le troisième cas humain de myiase gastro-intestinale identifié au Maroc et le premier cas décrit de myiase gastro-intestinale à *Drosophila melanogaster*. Cette observation s'individualise, d'une part, par sa localisation anatomique qui reste exceptionnelle, d'autre part par l'espèce incriminée. La prophylaxie reste le meilleur moyen pour lutter contre les myiases. Elle se base essentiellement sur l'enquête épidémiologique et le respect des règles d´hygiène générales.

## LIENS D'INTÉRÊTS

Les auteurs ne déclarent aucun lien et aucun conflit d'intérêts.

## CONTRIBUTION DES AUTEURS

Zaineb HAMMOUCH : conception de l’étude, prospection bibliographique, définition de la méthodologie, interprétation des résultats et rédaction du manuscrit.

Asmae EL ASSIL : conception de l’étude, prospection bibliographique et rédaction du manuscrit.

Mohamed LYAGOUBI : approbation de la version finale soumise à publication.

Sara AOUFI : supervision de l’étude, confirmation de l'exactitude et de l'intégrité des données, approbation et validation de la version finale soumise à publication.
